# Parameters of visual processing abnormalities in adults with body image concerns

**DOI:** 10.1371/journal.pone.0207585

**Published:** 2018-11-26

**Authors:** Sakshi Dhir, Hamish S. Ryan, Erin L. McKay, Matthew E. Mundy

**Affiliations:** School of Psychological Sciences and Monash Institute of Cognitive and Clinical Neurosciences, Monash University, Clayton, Victoria, Australia; University of Rome, ITALY

## Abstract

Body dysmorphic disorder (BDD), at the extreme end of the body image concern (BIC) spectrum, is thought to be associated with a local (detail-focussed) visual processing bias. Given that the inversion of a stimulus disrupts holistic processing and demands detail-specific attention, this perceptual bias is characterised by superior processing of such inverted stimuli. This study examined the processing bias, via a body-inversion discrimination task, of 26 participants with non-clinical, high-BIC (Dysmophic Concern Questionnaire (DCQ) scores between 11–19) and 26 participants with low-BIC (DCQ scores between 0–4). This study also explored the impact of varying stimuli presentation durations and discrimination difficulties during the inversion task on visual processing. As hypothesised, compared to those with low-BIC, participants with high-BIC demonstrated superior accuracy when discriminating between images of inverted bodies, indicating a local processing bias. Also as hypothesised, this local processing bias selectively manifested only when stimuli were presented for longer durations and at higher discrimination difficulties, revealing the parameters of this, potentially conscious, processing tendency. Consistent with previous research, this study identified a local processing bias in those with high BIC, which may be a predisposing factor for developing BDD. In turn, identifying the parameters (stimulus exposure and stimulus complexity) in which the local bias manifests has implications for future interventions aiming to reverse this perceptual abnormality.

## Introduction

### Body image concern

Body image concern (BIC) is becoming increasingly problematic in Western societies and is a common stressor among young adults [1]. Body image can be defined as a multi-dimensional phenomenon involving an individual’s subjective and malleable perceptions and feelings about their physical appearance [2–4]. Additionally, given that individuals vary in their level of body dissatisfaction, body image exists on a continuum ranging from healthy to unhealthy [5–6]. At the extreme end of the body image concern continuum, where an individual potentially experiences intense appearance disturbances, exists a clinical diagnosis of body dysmorphic disorder (BDD) [7].

### Body dysmorphic disorder

The Diagnostic and Statistical Manual of Mental Disorders [8] defines BDD as an excessive preoccupation with an imagined or exaggerated flaw in appearance. BDD has a prevalence rate of approximately 1.7% [9] and is associated with significant morbidity including a higher risk of psychiatric hospitalisation and high co-morbidity rates with other Axis 1 disorders [10–11]. Thus, investigation of risk factors and etiology is a critical area of research.

A high level of BIC in those with BDD is a defining marker of the disorder. In fact, research has shown that high BIC has a greater association with BDD even when compared to Bulimia and Anorexia Nervosa [12]. Additionally, behavioural models and neurocognitive research has proposed potential visual processing abnormalities in those with BDD. These processing abnormalities are in the form of a predisposition to pay excessive attention to specific details of stimuli [13–18]. Thus, there is potential for identifying these processing abnormalities as a possible risk factor for developing BDD. Given that those with high BIC are ‘at risk’ of BDD, examining the role of visual processing in these individuals is potentially valuable for investigating perceptual abnormalities as a risk factor.

### Abnormal visual processing

Visual perception generally involves a balance between global and local processing. Global processing refers to perceiving the ‘whole’ stimulus and is characterised by mental ‘exemplars’ of commonly encountered stimuli, which aid in recognition [19]. For example, a face is generally perceived globally based on schematic information of where typical facial features (eyes, mouth, nose) are positioned in relation to each other. On the other hand, local processing involves attention to detail-specific features of a stimulus [19]. It has been suggested that, as compared to healthy individuals [20–21], those with BDD potentially have a disordered imbalance between local and global processing, in which they overuse local processing whilst lacking in global perspective. This is thought to contribute to their abnormal, detail-specific fixation on appearance related stimuli. In particular, the processing imbalance may invoke the over-attention that those with BDD pay to their own imagined or exaggerated flaws [22–23].

The imbalance of global vs. local processing in those with high BIC and/or BDD has been commonly examined via stimulus inversion effects. This involves a task in which an initially upright image is rotated 180° from it’s typical orientation to become inverted [24]. Whilst the inversion effect (slower and less accurate processing when stimuli becomes inverted) is prominent in healthy individuals [24], studies have shown that it is weaker or non-existent for those with high BIC. This represents a perceptual deviation in these individuals. For example, research focusing on the inversion effect for faces, bodies and scenes has found that undergraduate university students with high BIC are able to discriminate inverted stimuli faster (faces and bodies) and more accurately (bodies and scenes) than those with low BIC [6]. This weakened inversion effect has been shown to increase simultaneously with BIC, such that, those with greater body dissatisfaction show superior visual processing of inverted stimuli [25]. Whilst these studies [6,25] identified those with varying levels of BIC in a healthy population using the Dysmorphic Concern Questionnaire (DCQ) [26], processing abnormalities, in the form of diminished inversion effects, have also been extended to clinical populations of those diagnosed with BDD [17,27]. Therefore, research has proposed a visual processing abnormality in those who have high BIC and those who have been clinically diagnosed with BDD.

However, many of these studies have used only facial stimuli when examining the inversion effect. This has been supported by research suggesting that the inversion effect weakens or disappears for headless body images, highlighting the significant role that faces play [28, 29, 30]. Nevertheless, this has not been well replicated, as other studies have shown substantial and similar inversion effects for both faces and headless bodies [6, 31, 32]. Similarly, literature has also suggested that bodies, like faces, are processed holistically and result in similar inversion effects [33, 34, 35]. Furthermore, global processing is based on mental ‘templates’ of commonly encountered stimuli, which then become disrupted when the inversion occurs. These mental ‘templates’ have been formed due to the frequency at which humans encounter stimuli such as faces and bodies on a daily basis [34]. Therefore, as bodies and faces are generally processed at the same frequency (it is rare to process a face without a body, vice versa), they should be equally salient in forming such mental ‘templates’ and, consequently generating the inversion effect. Regardless of this rationale, there is a deficiency in research focusing solely on the processing of body stimuli, as compared to the numerous studies on just facial recognition.

Several studies [6,17,25,27] examining perceptual abnormalities have identified the presence of a local processing bias in those with high BIC and/or BDD. It has been proposed that when the inversion occurs and the configuration of the stimulus (e.g. eyes typically located above nose) is disrupted, a changeover to local processing is swiftly demanded [28]. However, given the potential local bias in those with high BIC and/or BDD, these individuals are unaffected as they are already engaged in detail-specific processing. Consequently, the typical processing delay or adjustment due to the change from global to local processing does not impact those with high BIC and/or BDD. However, within the low BIC or healthy population, facial and body stimuli are typically processed holistically. Thus, the inversion of the stimulus results in an adjustment period as the mental ‘template’ used for global processing is disrupted. This adjustment period is characterised by slower and less accurate processing (the inversion effect). Therefore, reduced susceptibility to the inversion effect in individuals with BDD and/or high BIC epitomises the perceptual deviation that they experience from the typical normative processing.

Many of the studies proposing the presence of a perceptual abnormality have used clinical samples of those diagnosed with BDD [15,17–18,27,36]. However, the use of clinically diagnosed participants creates uncertainty regarding whether perpetual abnormalities are a predisposing risk factor or a consequence of the disorder. If these abnormalities are a risk factor, there is vital early-detection, prevention and early-intervention significance. Longitudinal research in a non-clinical, ‘at risk’ population would be ideal in determining whether this combination of body image concerns and visual processing abnormalities precedes the development of BDD. However, a cross-sectional study using a sample of those ‘at risk’ of BDD, such as those with non-clinical high BIC, is a useful step for first establishing a link between perceptual abnormalities and high BIC.

### Parameters of the processing bias

#### Stimuli presentation time

Conversely, some research [37–38] has challenged the presence of a local processing bias in those with BDD and/or high BIC. For example, Monzani [38] used a number of measures, including the face inversion task, to demonstrate that visual processing of those with BDD did not differ from healthy controls. Specifically, this study did not support the presence of a local bias or impaired holistic processing in those with BDD, which generally presents in the form of diminished inversion effects. However, this study presented stimuli during the inversion task for an unusually short period of time. Pairs of stimuli were presented, either upright or inverted, for a short duration of 250ms followed by a gap of 1000ms. Duncum [37] also found no perceptual deviation in those with high BIC, measured via an inversion task. Similarly, this study also consisted of an initial short presentation time of 500ms. These findings potentially suggest that when stimuli are presented for brief durations (under 500ms), all individuals may only have enough time to process holistically. Thus, discrepancies in research have been found and the inversion effect is not always shown in those with high BIC and/or BDD. This may be attributable to the conditions necessary, particularly exposure to stimuli, for the bias to manifest. For example, it has been suggested that the local processing bias in those with BDD and/or high BIC may not be an automatic way of processing [39]. Instead, this superior ability to fixate on particular details may be more of a conscious bias, which only appears in stimuli exposure durations long enough for the maladaptive perception to begin.

Identifying the point at which this processing bias manifests in those with high BIC and/or BDD has important implications. For example, this has significant potential for future interventions aiming to reverse the abnormality by inducing global processing. In this case, it would be potentially effective to target the perceptual sequence at a stage before the local bias manifests, highlighting the importance of identifying this particular point. However, only 2 studies [17,32] have directly explored the role of stimuli presentation duration and adequate replication is required. Feusner [17] compared the inversion effect in a clinical population and controls during short (500ms) and long (5000ms) stimuli presentation durations, and only found a local processing tendency in those with BDD during the long presentation time. Similarly, Odem-Lim [39] used an ‘at risk’ population of those with high dysmorphic concern and found that this group only paid greater attention to facial information when it was presented for long (1000ms), as compared to short (200ms), durations. Additionally, both these studies have focused on only facial stimuli, whilst neglecting body stimuli. Thus, further research is required to clarify the unidentified point in the visual sequence at which the potential local processing bias manifests in those with high BIC.

In order to clarify the point in the visual sequence that the potential processing bias manifests, future research should focus on exploring this bias at specific stimuli exposure durations. Whilst research has established that this bias may not exist at stimuli durations of 500ms [37], it has been shown at durations of 650ms [6]. Thus, it is likely that the bias may manifest between this timeframe of 500-650ms of stimuli exposure. It would be useful to examine a presentation duration within this parameter, such as 600ms, in order to further clarify the point at which the processing bias is instigated. Additionally, comparing stimuli presentation durations that are longer (such as 900 and 1200ms) and shorter (such as 300ms) than the parameters in which research [6, 25,17] has indicated that this bias exists would be useful in exploring whether this processing tendency decreases or increases with greater exposure to stimuli.

#### Stimuli discrimination difficulty

Additionally, there is also uncertainty around whether the local bias found in those with high BIC and/or BDD has a relationship with the degree of stimuli complexity. Leder and Bruce’s [40] facial differentiation task explored the impact of different stimuli complexity demands on perception. This study compared two stimuli situations: (1) when the faces could only be differed in terms of specific information (high complexity stimuli) and (2) when the faces could only be differed from each other holistically (low complexity stimuli). Changes in the second condition engaged the participant in global processing due to differences in very low spatial frequencies, such as facial shape. The first condition, however, engaged the participant in local processing. This local processing was demanded in the first condition due to high spatial frequency information, such as differences in shades of specific facial (eyes, nose, mouth) features. This study found that the inversion effect did not occur when the stimuli could only be discriminated locally because this potentially stimulated the viewer to engage in detail-specific processing from the onset. These findings suggest that presenting difficult to distinguish stimuli with high spatial frequencies, which potentially demand detail-specific processing, possibly reduces the inversion effect. Given that those with high BIC may already have a local processing bias, such complex stimuli processing demands are likely to have an even greater impact on reducing susceptibility to the inversion effect in these individuals.

Interestingly, a significant proportion of the research proposing a local bias has used very difficult to distinguish stimuli during the inversion task. These studies [6,17,25] have all had the optimal conditions, in terms of stimuli presentation time and stimuli discrimination difficulty, for those with high BIC to show a local processing tendency. More specifically, they have all used long presentation times and very difficult to distinguish stimuli during the inversion task. Those with high BIC possibly have a predisposition to process locally, thus, presenting difficult to distinguish stimuli that demand detail-focus may further precipitate this bias and contribute to the diminished inversion effects. This questions whether the same diminished inversion effects would be present in those with high BIC if pairs of images were easier to discriminate, demanding less focus on detail. However, research has not previously explored the impact of differing levels of stimuli discrimination difficulty on the local processing bias in those with high BIC. Exploring this gap in literature could potentially challenge the notion of a robust local processing bias in those with high BIC.

### The present study

In response to the identified gaps, this study will manipulate the stimuli conditions during a discrimination-inversion task to explore the parameters in which the proposed local processing bias manifests in those with high BIC. Simultaneously, limitations of previous research will be addressed by using a non-clinical sample of those with high BIC and by using images of bodies as stimuli for the inversion task.

Firstly, this study will aim to examine whether participants with high BIC, as compared to those with low BIC, have a local visual processing bias indicated by diminished inversion effects. Secondly, this study will aim to explore whether varying stimuli presentation time and discrimination difficulty, during the inversion task, has an impact on this possible local bias in those with high BIC.

Research has previously found that those with high BIC [6,25] and BDD [17,27] show diminished inversion effects indicative of a local processing bias. Thus, it is hypothesised that participants with high BIC, compared to those with low BIC, will show superior accuracy and reaction time when discriminating between inverted body stimuli (diminished inversion effects), indicating a local processing bias.

Additionally, research using the inversion task has suggested that those with high BIC are more likely to show a local processing bias in longer, as compared to shorter, stimuli presentation times [17]. Thus, it is hypothesised that the local processing bias in those with high BIC, indicated by diminished inversion effects, will only manifest during longer stimuli presentation times. More specifically, it is expected that the perceptual deviation will manifest, and become increasingly pronounced, when stimuli is presented for 600, 900 and 1200ms durations. However, it is expected that this effect will not be present when stimuli are presented for 300ms.

Furthermore, the impact of stimuli complexity on the local bias hasn’t yet been explored. Nevertheless, most of the research that has identified this local processing bias in individuals with high BIC has used very difficult to distinguish stimuli [6,17,25]. Thus, it is hypothesised that the local processing bias in those with high BIC, indicated by diminished inversion effects, will only manifest when stimuli more difficult to discriminate, with higher levels of difficulty determined by greater detailed similarity between the images.

## Method

### Participants

Participants were recruited via poster advertising at Monash University, Clayton Campus, Victoria and through online social media advertising. Participants completed an online screening questionnaire, which included an electronic version of the Dysmorphic Concern Questionnaire (DCQ). The study was approved by the Monash University Human Research Ethics Committee (CF14/750–2014000297).

The questionnaire received a total of 350 completed responses. Those who were below 18, did not have normal or corrected to normal vision, self-reported a previous diagnosis of an eating disorder or BDD, self-reported an acquired brain injury (ABI) or self-reported taking any psychotropic medications were excluded from the study. Email and telephone invitations to participate in the behavioural component of the study were sent out to 52 participants with the lowest scores on the DCQ within the sample (DCQ scores ranged from 0–4; 26 declined or did not respond) and 58 participants with the highest scores on the DCQ within the sample (DCQ scores ranged from 11–19; 32 declined or did not respond).

The final sample consisted of 4 males (*M*_age_ = 31, *SD* = 15.39) and 22 females (*M*_age_ = 22, *SD* = 1.24) in the low BIC group (*M*_BIC_ = 2.08, *SD* = 1.16) and 8 males (*M*_age_ = 19, *SD* = 3.16) and 18 females (*M*_age_ = 22, *SD* = 4.48) in the high BIC group (*M*_BIC_ = 14.08, *SD* = 2.65). Participation was incentivised by placing all those who completed the online questionnaire in a draw to win 1 of 4 $50 gift vouchers and those who completed the behavioural in-laboratory component of the study were compensated $40 for their time.

### Materials

*Qualtrics* hosted the online questionnaire. After the explanatory statement, the questionnaire collected demographic information including age, gender, vision, prior diagnosis of BDD or eating disorders, ABI status, whether the participant was taking any psychotropic medication and an email address and/or phone number. This information was used to assess whether respondents met the eligibility criteria to participate, to invite them to the second part of the study and to reward the participation incentive. The questionnaire then included an electronic version of the DCQ.

#### The Dysmorphic Concern Questionnaire (DCQ)

The 7-item DCQ [26] is a self-report measure of dysmorphic concern. Participants were required to indicate the ways in which they have perceived their body using a 4-point Likert scale, ranging from 0 (not at all) to 3 (much more than most people). The scale included questions such as “Have you ever: Spent a lot of time worrying about a defect in your appearance / bodily functioning?” and “Have you ever: Spent a lot of time covering up defects in you appearance/bodily functioning?”. The DCQ is scored by summing all the item responses together, resulting in a range of scores from 0 to 21 with higher scores corresponding to higher levels of BIC. This scale may be used as a screening tool for BDD, with scores above 9 representing clinically concerning levels of body dysmorphic concern [41]. Thus, the high BIC group within this study had DCQ scores ranging between 11 and 19, whilst those with current or past clinical diagnoses of BDD were excluded. However, it is important to note that some authors have recommended a higher cut-off (≥14) for greater sensitivity and specificity for correct diagnosis when working with BDD patients [42]. The DCQ has good internal consistency (α = .88) [26] and discriminant validity with BDD [43], making it an appropriate measure for this study.

#### Stimuli

**Fig 1:**
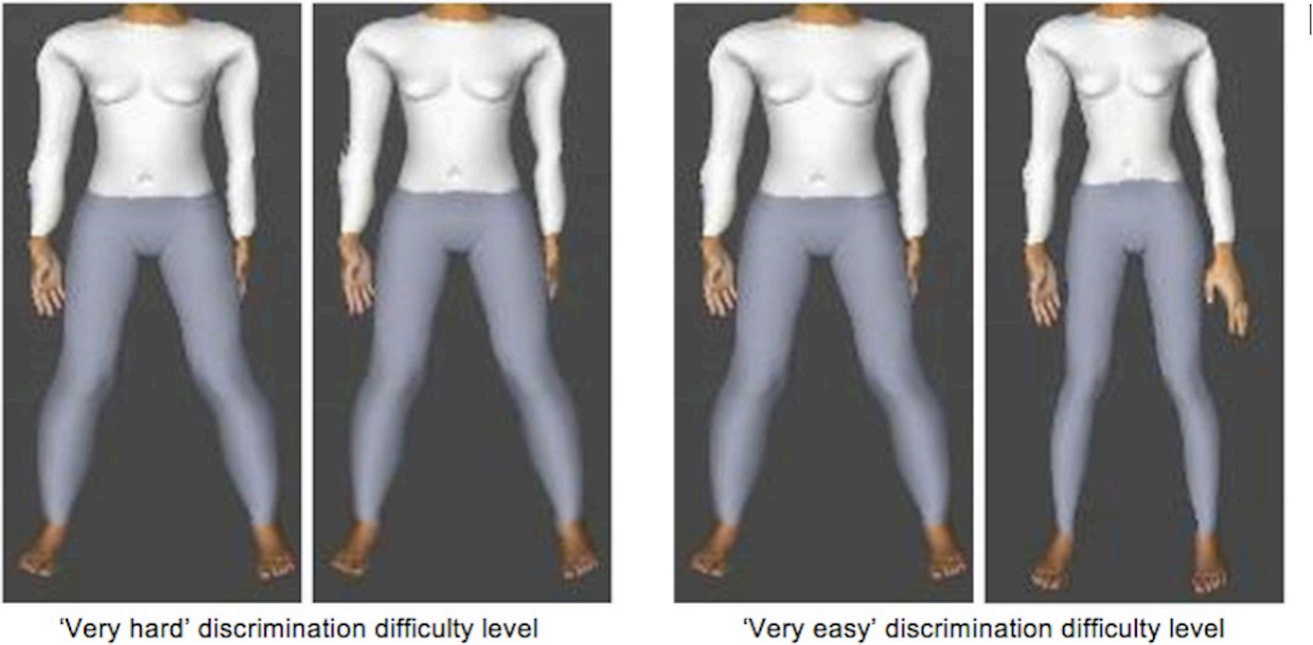
Examples of body stimuli at the ‘very hard’ and ‘very easy’ discrimination difficulty levels.

The in-laboratory behavioural component of this study involved the presentation of upright and inverted pairs of body images, which participants were instructed to discriminate between. The software HumanCAD [44] was used to create the body stimuli, which varied in terms of postures and shapes. An equal number of male and female pairs of fully clothed body images were created. The Morpheus Photo Morpher [45] software was used to morph the images together, resulting in 4 different discrimination difficulty levels (very easy, easy, hard, very hard). The morphing process merges the original two images together to create a continuous sequence of change across all image parameters that differ between the two original images (e.g., body shape, size, limb position, orientation etc.). The difficulty level was determined by how close together the original pairs of images (e.g., body A and body B) were on the morph continuum, with very easy at 70% body A and 30% body B, easy at 65% body A and 35% body B, hard at 60% body A and 40% body B and very hard at 55% body A and 45% body B. Examples of the stimuli at the ‘very hard’ and ‘very easy’ discrimination difficulty levels are shown in Fig 1.

Consistent with previous similar studies [6,37], the dimensions for all images on screen were 15 X 12 degrees of visual angle (height x width). The behavioral task ran on an IBM compatible PC and the software *Presentation* [46] was used to present stimuli and record reaction time (RT) and accuracy for discrimination between pairs of stimuli.

### Procedure

Participants initially completed the online questionnaire from which their DCQ scores were calculated. Those with scores within the low (DCQ scores < 5) and high (DCQ scores > 10) ranges were invited via email or telephone to take part in the behavioural experiment at Monash University, Clayton. When participants came into the laboratory, they were first provided with an explanatory statement and were asked to sign a consent form. Then they began the behavioural experiment, which involved a discrimination task where pairs of the body stimuli were presented either upright or inverted. Participants were seated approximately 70cm away from a 24 inch PC monitor and were instructed to indicate whether successive pairs of body stimuli were the same or different using the mouse (right click for same and left click for different). The behavioural experiment was conducted in 2 separate blocks to ensure that participants took part in all conditions of the independent variables, as shown in Table 1.

**Table 1:** Design of the behavioural experiment and variables of interest.

Block	Independent Variables				Dependent Variables	
	Presentation time	Discrimination difficulty	Orientation	BIC	Accuracy	RT
1	(1) Very long (2) Long (3) Short (4) Very short	(1) Very hard	Upright Inverted	High Low		
2	(1) Very long	(1) Very hard (2) Hard (3) Easy (4) Very easy	Upright Inverted	High Low		

#### Block 1

Block 1 manipulated stimuli presentation time. A control discrimination difficulty level of ‘very hard’ was used, however, each trial consisted of different stimuli presentation times. The trials included the ‘very long’ condition with a stimuli presentation time of 1200ms, the ‘long’ condition with a stimuli presentation time of 900ms, the ‘short’ condition with a stimuli presentation time of 600ms and the ‘very short’ condition with a stimuli presentation time of 300ms. A blank screen followed each trial for 500ms before the next trial began. Each condition was presented randomly. The participant’s response period began 100ms into the second stimuli presentation period and ended after 4s. Trials with no responses were discarded.

#### Block 2

Block 2 manipulated stimuli discrimination difficulty. All trials in the second block consisted of a control presentation time of 1200ms, followed by a blank screen for 500ms and then a second stimulus for 1200ms. However, in this block, the similarity between the pairs of stimuli was manipulated to create different levels of discrimination difficulty. The levels included ‘very hard’, ‘hard’, ‘easy’ and ‘very easy’. The trials were presented randomly. The overall design of the behavioural experiment is presented in Table 1.

Each of the blocks contained 8 stimuli pairs with 4 pairs presented upright and 4 inverted at 180°. Each stimuli pair was presented 6 times in each block. Of the 48 trials in each block, 24 pairs were non-matching (the original stimulus and it’s morphed version), thus required participants to respond ‘different’. The rest of the 24 trials were matching (the stimulus and it’s exact copy), thus required participants to respond ‘same’. The behavioural task for this study took approximately 25 minutes. However, participants also completed behavioural tasks for another 2 studies, resulting in the total participation time of approximately 1.5 hours.

## Results

Data were analysed using SPSS statistics version 21 (IBM, Australia) and an alpha level of .05 (two-tailed) was used for all statistical analyses. Accuracy scores were calculated as a percentage of correct responses out of the total number of trails in each block. Reaction time (RT) was only analysed for the correct responses. Each participant obtained both accuracy and RT scores for block 1 (manipulated stimuli presentation time) and block 2 (manipulation stimuli discrimination difficulty). Thus, 4 ANOVAs were conducted on 4 independent sets of data and follow up analyses were conducted for significant effects. Prior to analysis, data was cleaned and assumptions were inspected. There were 5 outliers with z-scores outside the -3.29 to 3.29 range, which were winsorized to next greatest non-outlier values [47]. The analysis was assumed to be robust to violations of normality and homogeneity of variance assumptions due to equal groups and a moderate sample size [46]. Mauchly’s test (*p* > .05) indicated that the assumption of sphericity was met for 3 out of 4 ANOVAs [39]. The ANOVA that violated the assumption of sphericity was corrected via the Huynh-Feldt estimate [46].

### Accuracy analysis

#### Overall inversion effects

Firstly, a 2 (BIC) x 4 (presentation time) x 2 (orientation) mixed factorial ANOVA was conducted to explore differences in processing accuracy as a function of BIC (between subjects factor), stimuli presentation time (within-subjects factor) and stimulus orientation (within-subjects factor). There was a significant main effect for stimuli orientation, *F*(1,50) = 15.78,*p* < .01,*partial n*^2^ = .85. The upright pairs of images resulted in significantly higher accuracy scores (*M* = 78.45, *SE* = .60) 95% BCa [77.25, 79.65], as compared to the inverted pairs of images (*M* = 65.23, *SE* = .50) 95% BCa [64.22, 66.23]. This indicated the presence of an overall inversion effect.

#### Diminished inversion effects

There was a significant interaction between stimuli orientation and BIC group for accuracy scores, *F* (1,50) = 38.91,*p* < .01,*partial n*^2^ = .44. Simple effects analysis, with a Bonferroni adjusted alpha level, revealed no significant differences between the groups for accurately discriminating between upright body stimuli, *F* (1, 412) = 1.35, *p* = . 245. However, there was a significant difference in accuracy scores between the groups when discriminating between inverted body stimuli, *F* (1,412) = 48.20,*p* < .01,*partial n*^2^ = .11. The high BIC group showed significantly higher accuracy scores than the low BIC group when discriminating between inverted body stimuli, *M*_*diff*_ = 8.43,*SE* = 1.22,*p* < .01,95% CI [6.05,10.82]. Further independent samples t-tests indicated that greater accuracy in the upright as compared to inverted condition, indicative of the inversion effect, was statistically significant for both the low (*M*_*diff*_ = 18.14,*SE* = .98) and high (*M*_*diff*_ = 8.30,*SE* = 1.41) BIC groups individually, *t*(192.16) = 18.46, *p* < .01, 95% CI [16.21, 20.08], *t*(194.82) = 5.89, *p* < .01, 95% CI [5.52, 11.08], respectively. Additional t-tests on participant difference scores in processing upright vs. inverted stimuli between high and low BIC groups revealed that the difference in inversion effects between the groups was significantly significant, *t*(50) = 4.74, *p* < .01, 95% CI [7.31, 18.07], *d* = 1.31. Thus, those in the high, as compared to low, BIC group showed diminished inversion effects. See Table 2 for estimated marginal means and confidence intervals for this interaction.

**Table 2:** Estimated marginal means and confidence intervals of accuracy scores for the interaction between BIC group and stimuli orientation.

BIC Group	Orientation	*M (SE)*	95% Cl
Low	Upright	79.15 (.84)	[77.46, 80.85]
	Inverted	61.01 (.70)	[59.59, 62.43]
High	Upright	77.74 (.84)	[79.05, 79.44]
	Inverted	69.44 (.71)	[68.02, 70.86]

Cl = confidence interval. SE = standard error. M = mean. BIC = body image concern.

#### Stimuli presentation time

**Fig 2:**
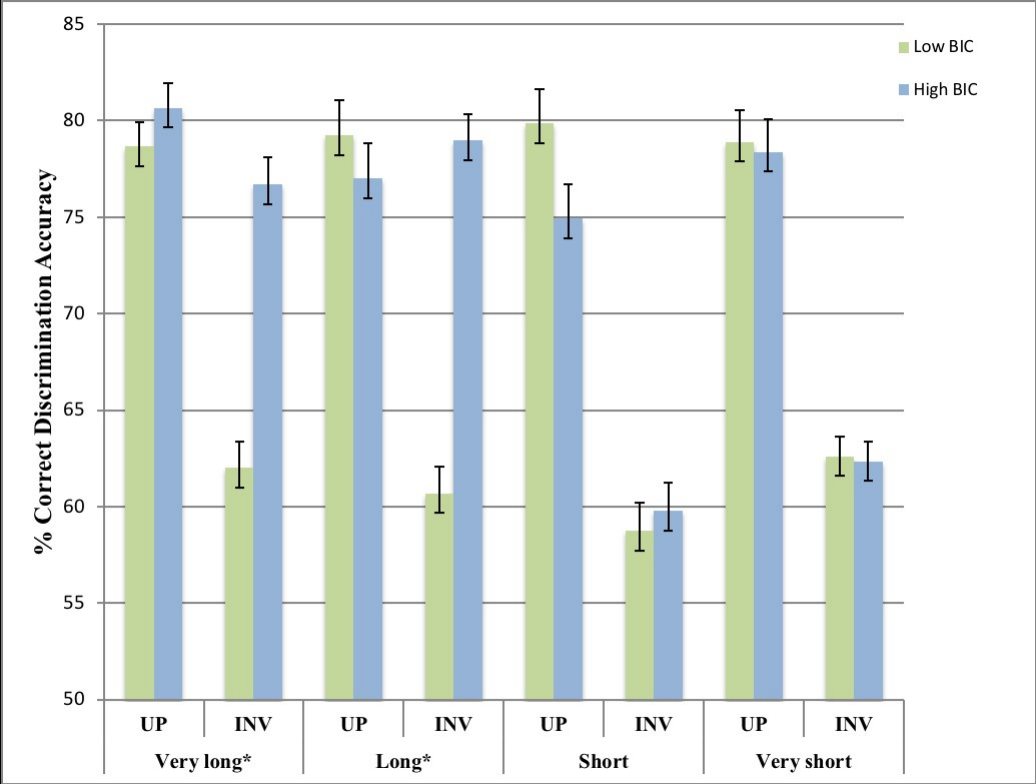
Accuracy rates (%) for upright (UP) and inverted (INV) stimuli discrimination across presentation durations. Error bars indicate *SEM*. * *p* < .01.

There was a significant interaction between BIC group, stimuli presentation time and stimuli orientation, *F*(3,150) = 8.59,*p* < .01,*partial n*^2^ = .15. Simple effects analysis, with a Bonferroni adjusted alpha level, revealed significant differences between the groups when accurately processing the inverted stimuli during the very long and long stimuli presentation time conditions, *F*(1,400) = 47.89,*p* < .01,*partial n*^2^ = .11, *F*(1,400) = 74.04,*p* < .01,*partial n*^2^ = .16, respectively. The high BIC group, as compared to the low BIC group, had significantly higher accuracy scores when discriminating between inverted stimuli in the very long *M*_*diff*_ = 14.69,*SE* = 2.12,*p* < .01,95% *CI* [10.52,19.87] and long, *M*_*diff*_ = 18.27,*SE* = 2.12,*p* < .01,95% *CI* [14.10,22.44], stimuli presentation time conditions. Additional t-tests on participant difference scores in processing upright vs. inverted stimuli between BIC groups revealed that the inversion effect was significantly diminished in the high, as compared to low, BIC group in the very long, *t*(50) = 4.74, *p <* .01, 95% CI [7.31, 18.07], *d* = 1.31, and long, *t*(50) = .10, *p* < .01, 95% CI [13.70, 27.30], 1.64, stimuli presentation conditions. However, there were no significant differences between the groups when accurately processing inverted stimuli during the short and very short stimuli presentation time conditions, *F*(1,400) = .24, *p* = .625, *F* (1, 400) = .02, *p* = .899, respectively. Additional independent samples t-tests on participant difference scores in processing upright vs. inverted stimuli between BIC groups revealed that greater processing of upright as compared to inverted stimuli (inversion effect) was not significantly different in the high, as compared to low, BIC groups during the short, *t*(50) = 1.85, *p* = .07, and very short, *t*(50) = .084, *p* = .93, stimuli presentation time conditions. Thus, diminished inversion effects in the high BIC group were only evident in the longer stimuli presentation times (see Fig 2).

#### Stimuli discrimination difficulty

**Fig 3:**
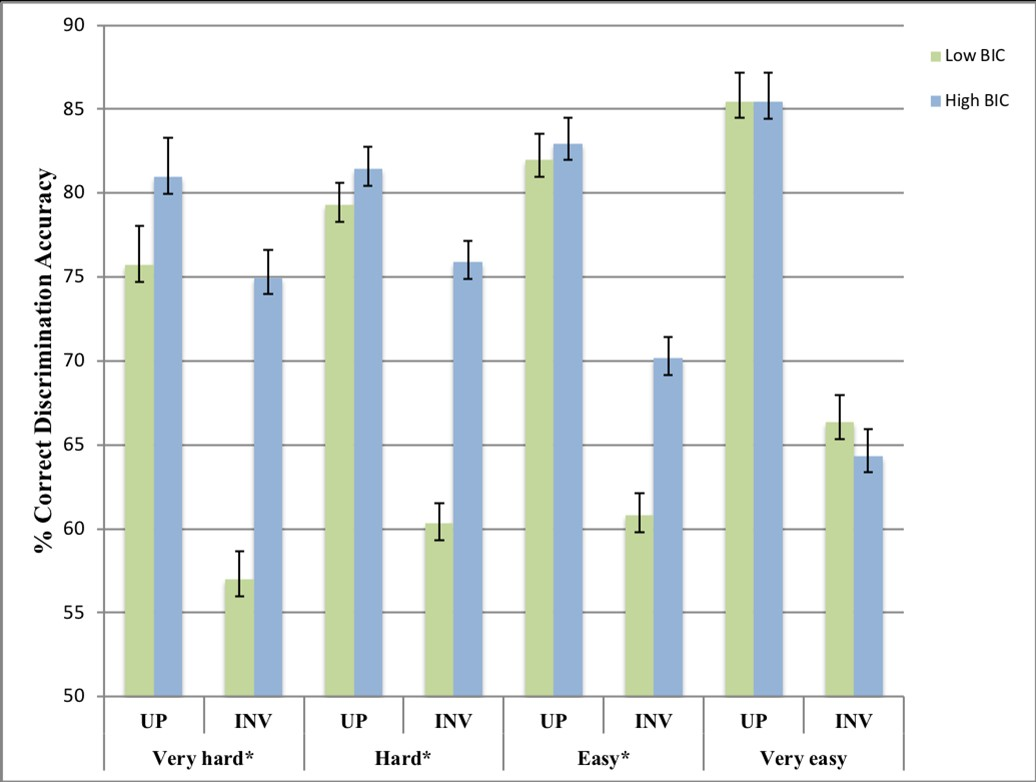
Accuracy rates (%) for upright (UP) and inverted (INV) stimuli discrimination across discrimination difficulties. Error bars indicate *SEM*. * *p* < .01.

Secondly, a 2 (BIC) x 4 (difficulty level) x 2 (orientation) mixed factorial ANOVA was conducted to explore differences in processing accuracy as a function of BIC (between-subjects factor), stimuli discrimination difficulty (within-subjects factor) and stimuli orientation (within-subjects factor). There was a significant interaction between stimuli discrimination difficulty, stimuli orientation and BIC group across accuracy scores, *F*(2.74,137.11) = 6.40,*p* < .01,*partial n*^2^ = .11. Simple effects analysis, with a Bonferroni adjusted alpha level, revealed significant differences in accuracy scores between the high and low BIC groups when distinguishing between inverted stimuli during the very hard, *M*_*diff*_ = 17.96,*SE* = 2.29,95% *CI* [13.47,22.46], hard, *M*_*diff*_ = 15.58,*SE* = 2.29,95% *CI* [20.07,−11.01] and easy, *M*_*diff*_ = 9.35,*SE* = 2.29,95% *CI* [−13.84,−4.85], discrimination difficulty conditions, *F*(1,400) = 61.74,*p* < .01,*partial n*^2^ = .13,*F* (1,400) = 46.44,*p* < .01,*partial n*^2^ = .10,*F* (1,400) = 16.72,*p* < .01,*partial n*^2^ = .04, respectively. Additional t-tests on participant difference scores in processing upright vs. inverted stimuli between BIC groups revealed that this inversion effect was significantly diminished in the high, as compared to low, BIC group in the very hard, hard, and easy discrimination difficulty conditions, *t*(50) = 4.02, *p <* .01, 95% CI [6.37, 19.09], *d =* 1.11, *t*(50) = 6.26, *p* < .01, 95% CI [9.12, 17.73], *d =* 1.11, *t*(41.75) = 2.91, *p <* .005, 95% CI [2.57, 14.20], *d* = .81, respectively. However, there were no differences in accuracy scores between the BIC groups when distinguishing inverted stimuli during the very easy discrimination difficulty condition, *F* (1, 400) = .77, *p* = .382. Additional t-tests on participant difference scores in processing upright vs. inverted stimuli between BIC groups revealed that greater processing of upright as compared to inverted stimuli (inversion effect) was not significantly different in the high, as compared to low, BIC groups during the very easy discrimination difficulty condition, *t*(50) = -.72, *p* = .47. Thus, the high BIC group, as compared to the low BIC group, showed significantly lower/diminished inversion effects only during the very hard, *M*_*diff*_ = 12.73,*SE* = 3.16, hard, *M*_*diff*_ = 13.42,*SE* = 2.14, and easy, *M*_*diff*_ = 8.38,*SE* = 2.88, stimuli discrimination difficulty conditions (see Fig 3).

### Reaction time analysis

#### Inversion effects

Thirdly, a 2 (BIC) x 4 (stimuli presentation time) x 2 (stimuli orientation) mixed factorial ANOVA was conducted to examine the aforementioned relationships but across RT scores. This showed a non-significant main effect for stimuli orientation and a non-significant interaction effect between stimuli orientation and BIC group, *F* (1, 50) = .13, *p* = .717, *F* (1, 50) = .10, *p* = .754, respectively.

#### Stimuli presentation time

Additionally, there was a non-significant interaction effect between stimuli presentation time, stimuli orientation and BIC group, *F* (3, 150) = 2.12, *p* = .10.

#### Stimuli discrimination difficulty

Finally, a 2 (BIC) x 4 (stimuli discrimination difficulty) x 2 (stimuli orientation) mixed factorial ANOVA on RT showed a non-significant interaction effect between stimuli discrimination difficulty, BIC group and stimuli orientation, *F* (3, 150) = 1.69, *p* = .171.

## Discussion and conclusion

The current study investigated whether a local visual processing bias was present in a non-clinical sample of individuals with high BIC. The local processing bias in the high BIC group was indicated via a superior ability, as compared to the low BIC group, to discriminate between inverted images of bodies. The influence of stimulus presentation duration and stimulus discrimination difficulty during the body-inversion task on this perceptual bias was also examined. Consistent with previous research [48–49] an overall standard inversion effect was found for all healthy participants, as upright stimuli were processed more accurately than inverted stimuli. However, as hypothesised, this inversion effect was significantly weaker for those with high, as compared to low, BIC. This highlighted a processing deviation from global to local in these individuals. Also as hypothesised, this local processing bias in those with high BIC only manifested when stimuli were presented for longer durations and at greater discrimination difficulties within the inversion task.

There were no significant differences found in reaction time between the high and low BIC groups, potentially due to a trade off between accuracy and speed. It is suggested that participants are more likely to value achieving the correct response than reacting quickly, unless otherwise instructed. As completion speed was not addressed in participant instructions for this study, any conclusions made within the present study will be based on processing accuracy.

### Diminished inversion effects

The identified diminished inversion effects in individuals with high BIC is directly in line with previous research [6,25,17,27]. Also consistent with this previous research, the present study theorises that the diminished inversion effects are explained by the dominance of a local processing bias in those with high BIC. Prior brain imaging research has proposed neurological explanations for this perceptual deviation [15,18]. It has been suggested that those with BDD experience hyperactivity [18] in neural areas that are involved in detail-specific processing and hypoactivity [15] in areas that are associated with low-detail, configural processing. As a result, those with BDD may have a tendency to focus on details, whilst lacking holistic insight. Thus, according to this explanation, those with high BIC were already engaged in the detail-specific processing that was suddenly demanded by the presentation of the inverted stimuli. As a result, the high BIC group was safeguarded against the inversion effect due to a potential bias to process locally.

Additionally, the identified processing abnormality is consistent with the notion that those with BDD process visual stimuli in a piecemeal way [17]. It is likely that those with low BIC processed the upright images based on mental schemas of the structural shape of a typical body [19]. This ‘template’ then became unusable when inversion occurred, meaning that local processing was suddenly demanded, thus, resulting in a perceptual delay. However, it is likely that those with high BIC and/or BDD process stimuli, regardless of orientation, in a more piecemeal fashion than healthy controls. Therefore, this detail-focussed perceptual tendency resulted in similarly accurate processing of both the inverted and upright stimuli in individuals with high BIC.

Furthermore, the local processing abnormality is somewhat reflected in BDD’s symptomology. The proposed perceptual fixation in those with high BIC mirrors the scrutinising way that those with BDD view their own appearance. This theory is aligned with the findings of Beiharz [25], that BIC and the local processing bias increase simultaneously. These findings show that those with higher levels of BIC, a defining feature of BDD, have greater levels of a detail-specific processing fixation. A logical extension from this suggests that the local processing bias potentially primes individuals to engage in detail-analysis when viewing their own appearance, in turn, exacerbating body dissatisfaction. At the extreme end of this processing fixation, individuals may view their appearance with such scrutiny that they become completely preoccupied with imagined and/or exaggerated flaws. In turn, this maladaptive perception may result in an extreme level of BIC and a potential diagnosis of BDD. Therefore, as this current study found the presence of a local processing bias in a non-clinical, ‘at-risk’ sample of those with high BIC, it adds to the body of evidence that a visual processing bias may potentially precede a clinical diagnosis of BDD.

### Stimuli presentation time

The diminished inversion effects in the high BIC individuals were only present during the longer stimuli presentation durations. These results are consistent with research that has compared short and long stimuli presentation durations [17], research that has used longer stimuli presentations and found a perceptual bias [6,25] and research that has used shorter presentation durations and found no perceptual differences [37–38]. These findings indicate that a local processing bias in those with high BIC manifests only after viewing stimuli for a particular duration of time. A possible explanation for this extends from the global precedent effect, which suggests that all individuals, including those with high BIC, will naturally use automatic global processing when first exposed to a stimulus [50]. Therefore, those with high BIC may not have a predisposed tendency to always process locally. Instead, it is likely that this is potentially a more conscious perceptual bias, which manifests only when there is sufficient exposure to the stimulus. Similarly, Feusner, Moller et al. [17] suggested that those with BDD have a superior ability for visual screening and detail encoding. However, this processing advantage is only evident when these individuals have enough time to process the elements within the image. Future eye-tracking studies, such as those of Greenberg [51] and Toh [52], but with a focus on whether those with BDD fixate on details as opposed to holistic information would be useful.

Additionally, BDD symptomology could also explain the significance of stimuli presentation duration. Those with BDD typically make comparisons to their own appearance when viewing others [11]. Therefore, they may consciously fixate on the details of other appearance related stimuli in order to make these comparisons [17,39]. It is likely that only longer durations of exposure to stimuli allow for this detailed, comparative processing to take place. Thus, this may explain why the maladaptive processing in the high BIC group only occurred during longer stimuli presentation times.

The present study has found the longest, reported, stimuli exposure duration in which a processing bias in high BIC individuals does not manifest (600ms). Given that Mundy and Sadusky [6] reported the shortest stimuli exposure duration in which this bias has been shown (650ms), it is suggested that the potential processing bias is instigated at some stage between 600ms and 650ms when those with high BIC view stimuli. That said, the present study and that of Mundy and Sadusky [6] are similar, but not directly comparable in methodology. For example, Mundy and Sadusky [6] used images of faces and scenes as well as body stimuli, which may have provided participants with a greater level of detail to fixate on and in turn resulted in a more pronounced tendency to engage in local processing. Therefore, whilst this study has taken a step towards further clarifying the point in the visual sequence that a bias may manifest, it is likely that this may fluctuate depending on the type of visual stimuli. Nonetheless, future interventions could potentially target the range of 600-650ms of stimulus exposure to attempt to reverse the local processing bias. Therefore, this finding has significance for potentially directly addressing the perceptual abnormality in those with high BIC and/or BDD.

### Discrimination difficulty

This is the first study to find that those with high BIC only showed diminished inversion effects when stimuli were more difficult to distinguish. Those with low BIC showed increasingly lower discrimination accuracy as similarity between the inverted stimuli increased, whilst those with high BIC simultaneously demonstrated increasingly superior accuracy. Similar to Leder and Bruce’s [40] proposition that the inversion effect does not exist if the stimuli only engage detail specific processing, a potential explanation could be that the harder to distinguish stimuli instigated detail-focussed processing in those with high BIC. However, these stimuli processing demands may not have been complex enough to initiate local processing in the low BIC individuals, thus, explaining why controls still showed the inversion effect. This suggests that the potential local processing bias in those with high BIC may be particularly sensitive to complex stimuli processing demands.

Conversely, the high BIC group did not show diminished inversion effects during the ‘very easy’ discrimination condition. As the stimuli in this condition were more likely to be differentiated based on configural information (e.g. the general size of the body shapes), this may have only demanded global, ‘template’-based processing. This low-complex processing demand potentially overpowered the local processing bias in those with high BIC, resulting in these individuals being vulnerable to the inversion effect during this condition. These findings are inconsistent with fMRI research showing that those with BDD show abnormal left hemisphere hyperactivity, which results in a tendency to engage in detail processing even viewing low spatial frequency stimuli [17, 18]. It evident in these prior studies that the ‘low frequency’ images are still far more detailed than the images used in the ‘very easy condition’. This is potentially due to the presence of greater specific facial details (nose, mouth, eyes) in these studies using face stimuli, as compared to the extremely low frequency body images used in the ‘very easy’ condition in this study. This highlights that whilst individuals with high BIC are far more susceptible to local processing, they are still likely to holistically process stimuli with extremely global, low-complex processing demands.

In light of these findings, a logical extension suggests that those with high and low BIC have different thresholds for responding to stimuli processing demands. More specifically, those with low BIC are likely to process commonly encountered stimuli, such as bodies, globally. Thus, these individuals were vulnerable to the body-inversion effect, regardless of stimuli complexity. Comparatively, those with high BIC may require a far greater demand for global processing in order to deviate from their possible local processing tendency. Thus, this high tolerance against global processing demands may explain why these individuals only showed the inversion effect in the ‘very easy’ discrimination condition. However, as compared to those with low BIC, individuals with high BIC may require far less-complex stimuli demands to engage in local processing. This was evident as they showed diminished inversion effects, indicative of the local bias, as soon as stimuli gained any level of complexity above ‘very easy’. Therefore, as compared to those with low BIC, individuals with high BIC may have a greater sensitivity to process locally and a greater tolerance against global processing demands.

These findings suggest that the local processing bias in those with high BIC is more of a conscious effort, rather than an all-encompassing underlying tendency. As previously stated, those with BDD are likely to make comparisons to their own appearance when viewing images such as bodies [11]. It is possible that when these images don’t include detail-specific features, those with high BIC do not have anything meaningful to fixate on. Whereas, when these images include more meticulous features, which these individuals may normally use as comparison points, the local perceptual fixation is more likely to appear. In turn, it is possible that this bias only manifested during the harder stimuli discrimination conditions because this drew attention to the specific details of the body stimuli, which individuals with high BIC would normally constitute as comparison points. Thus, this abnormal processing is potentially a more conscious bias that maintains maladaptive perceptions related to high BIC and/or BDD pathology.

### Limitations

This study’s cross-sectional design limits the conclusions that can be made regarding the causal relationship between a local processing bias and BDD. Whilst a local processing bias in those with high BIC has been identified, it cannot be concluded that this precedes a diagnosis of BDD without a longitudinal study. Additionally, given that it is difficult to conclude that participants in the high BIC group were pre-clinical, we again cannot be confident that visual processing abnormalities precede a diagnosis of BDD. In fact, it is possible that this perceptual fixation may be a consequence of having a high BIC and may not eventuate to a diagnosis of BDD. Nonetheless, this study provides a strong rationale for future longitudinal research to address this limitation by exploring whether those with high levels of BIC and a local processing bias eventually experience the development of BDD symptoms. Using a longitudinal design to identify whether this bias is predictive of high BIC symptomology has early detection and intervention utility. It would also be useful for future research to compare those clinically diagnosed with BDD and those at risk to shed light on whether this perceptual bias is a predisposition which becomes increasingly pronounced at clinical levels.

Whilst the use of bodies in this study was to address a gap created by the large range of research that has used just faces as stimuli, this limits the generalisability results. Future research should aim to explore whether, as compared to body stimuli, similar patterns of visual processing also appear when viewing other types of stimuli such as faces. Additionally, the current study also did not include neutral stimuli as a control. This limits the generalisability of the results to non-appearance related, neutral stimuli. Future research should address this by using alternative paradigms, which don’t rely on specific appearance-related stimuli, such as Navon’s global-local paradigm [21],

A non-clinical sample was used to strengthen the body of evidence proposing that visual processing abnormalities could potentially be one of the risk factors for developing BDD. However, given that a DCQ score of above 9 has been identified as clinically concerning [34] and the high BIC group in this study scored between 11 and 19, it is possible that some individuals within the sample were simply undiagnosed and should have been excluded. Whilst this limitation should be considered when interpreting the results, it was not within the scope of this current study to undertake clinical assessments of all participants. Future research with a more stringent criterion for determining whether participants are ‘at risk’ or undiagnosed should aim to further clarify this relationship.

A further limitation is that, due to time constraints, this study did not control for additional participant variables that may have influenced visual processing. For example, negative mood [53], autistic traits [54] and eating disorder traits [55] have all been reported to have an association with a local processing tendency. Additionally, different personality traits, such as impulsivity [56], and particular personality disorders, such as obsessive-compulsive personality disorder [57], have also been linked to more local visual processing. Thus, future studies should attempt to control the influence of these variables.

The inversion effect has been proposed to be an indirect measure of holistic processing [58], thus it would be useful for future research to utilise a more direct measure of configural processing to replicate the current findings. Commonly used direct measures of configural processing include the composite task [59–61] and the part-whole task [62]. For example, in the composite task participants are presented with either 2 halves of different faces aligned together or just 1 face. Participants are asked to determine whether the halves are the “same” or “different”. The aligned face halves are designed to appear as one entity, and consequently make it difficult for the participant to disassociate between them. The original version of this task uses accuracy and reaction time estimates to measure holistic processing. It would be interesting to examine whether such direct measures of holistic processing produce similar results to the large body of research that has utilised the inversion effect.

### Broader implications

Whilst further research is required to determine the causal relationship between BDD and visual processing abnormalities, this study provides some evidence that those with pre-clinical levels of BIC have a possible local processing bias. The high BIC sample within this study is considered an ‘at risk’ group for developing BDD, therefore, this study’s findings have important significance for establishing the relationship between high BIC symptoms and visual processing. Although there may be many factors that contribute to the development of BDD, the identification of visual processing abnormalities in individuals with high BIC provides considerable rational for future longitudinal research to clarify whether this is a potential risk factor. This is an important future direction for research as it may improve early identification of those at risk of BDD via a potential visual perception task designed to measure local processing tendencies, such that assessing visual processing abnormalities could be one of numerous measures used to identify those at risk of BDD. Thus, research establishing the relationship between high BIC, a key feature of BDD, and visual processing biases provides a promising foundation for further exploring risk factors for BDD.

Additionally, the selective manifestation of the local processing bias in particular stimuli exposure and stimuli complexity cnditions raises some interesting considerations. Firstly, this provides evidence that the local processing bias is potentially a more conscious process, which may only manifest in situations that are similar to when those with high BIC and/or BDD engage in appearance evaluation. More specifically, those with high BIC and/or BDD may only engage in detail-focused processing when there is enough time to make appearance-comparisons (longer stimuli presentation times) and only when the stimuli demands are somewhat more detailed (greater discrimination difficulty). This means that the processing bias may be far more impressionable than previously thought, providing rational for a future intervention to manipulate this perceptual tendency in an attempt to combat BDD symptoms. Such an intervention should maximise potential effectiveness by considering the identified parameters in this study that must exist for a processing bias to manifest. For example, it would be advisable, based on the findings of this study, for a potential intervention to induce global processing between 600ms and 650ms after stimulus onset to ensure that perception is targeted before it becomes maladaptive. Addressing the abnormal processing patterns that those with BDD engage in could alter the preoccupation that they have with their own flaws and their extreme levels of body dissatisfaction.

### Concluding remarks

The present study identified a local visual processing bias in a non-clinical sample of individuals with high BIC. However, it was evident that this bias selectively only manifested when these individuals were exposed to stimuli for longer durations and when stimuli processing demands consisted of greater complexity. For the first time within this area of research, such specific parameters necessary for a local processing tendency to dominate were identified. These findings shed light on the conscious and malleable nature of the processing bias in those with high BIC.

It would be interesting to explore whether the parameters of the local processing bias are consistent across all levels of BIC. For example, it could be possible that the higher BIC is, the less time and stimuli complexity is required for this processing bias to manifest. Thus, future research should investigate the necessary conditions for a local processing bias to manifest across a wider spectrum of BIC. Examining whether the local processing bias is exhibited at a faster (shorter presentation time) and more common (less-complex stimuli) rate as BIC increases, may clarify whether a high severity of this processing abnormality precedes the development of BDD.

## Supporting information

S1 Data Dataset. (XLSX)

S2 Data ANOVA. (XLSX)

S3 Data Simple effect analysis. (XLSX)

S4 Data *t*-tests. (XLSX)
